# Association between renal function and co-infection with *Clonorchis sinensis* and *Helicobacter pylori*: a cross-sectional study

**DOI:** 10.1186/s12879-020-05616-0

**Published:** 2020-11-19

**Authors:** Weining Xie, Yuanjun Deng, Shengxin Chen, Yifeng Wu, Ye Li, Qinhe Yang

**Affiliations:** 1 Department of Infectious Disease, Guangdong Provincial Hospital of Integrated Traditional Chinese and Western Medicine, Foshan, 528200 China; 2 School of Traditional Chinese Medicine, Jinan University, Guangzhou, 510632 China

**Keywords:** *Clonorchis sinensis*, *Helicobacter pylori*, Renal function, eGFR

## Abstract

**Background:**

Studies have shown that liver fluke infections may be associated with kidney injury and that *Helicobacter pylori* (Hp) may be involved in the pathogenesis of kidney diseases. However, no studies have reported the relationship between co-infection with *Clonorchis sinensis* (Cs) and Hp and renal function. The aim of this study was to examine the relationship between co-infection with Cs and Hp and estimated glomerular filtration rate (eGFR) in a general population, and gender-related differences were also investigated**.**

**Methods:**

In the cross-sectional study, 4122 subjects from the Health Examination Center of Guangdong Provincial Hospital of Integrated Traditional Chinese and Western Medicine from January 2017 to December 2018 were enrolled. All participants underwent stool examination for the diagnosis of Cs infection and ^13^C-urea breath test (UBT) for the diagnosis of Hp infection. Participants were categorized into four groups: (1) co-infection with Cs and Hp group comprising 207 cases (Hp(+) + Cs(+) group), (2) Cs infection group comprising 1392 cases (Hp(−) + Cs(+)group), (3) Hp infection group comprising 275 cases (Hp(+) + Cs(−) group), and (4) non-infection group comprising 2248 cases (Hp(−) + Cs(−) group). Multiple linear regression analysis was performed to evaluate the relationship between co-infection with Cs and Hp and eGFR.

**Results:**

Hp infection without Cs infection was present in 6.67% (275/4122) of subjects, while Cs infection without Hp infection was present in 33.77% (1392/4122) of subjects. Co-infection with Hp and Cs were present in 5.02% (207/4122) of subjects. Median age of the participants was 43 years (IQR 35–51). Most of the participants were male (2955/4122, 71.69%). Median eGFR was 96.61 ml/min/1.73 m^2^ (IQR 85.05–106.24). Co-infection with Cs and Hp was negatively associated with eGFR after full adjusting (β = − 1.89, 95% CI: − 3.33 to − 0.45, *p* = 0.01). The relationship remained significant in females (β = − 9.37, 95% CI: − 11.60 to − 7.1, *p* < 0.001), but not in males.

**Conclusion:**

Our findings suggest that co-infection with Cs and Hp may be associated with reduced renal function in females, but not in males.

**Supplementary Information:**

The online version contains supplementary material available at 10.1186/s12879-020-05616-0.

## Background

Infection of *Clonorchis sinensis* (Cs) is mainly prevalent in Asian countries and regions, including South Korea, China, Northern Vietnam, and Russian Far East [1–3]. China has the largest population with Cs infection, which is estimated at 13 million [3–5]. *Helicobacter pylori* (Hp) is the most common chronic bacterial infection in humans and is related to various gastrointestinal diseases, such as gastritis, peptic ulcer, gastric cancer, and extranodal marginal zone lymphoma of mucosa-associated lymphoid tissue [6, 7]. The prevalence of Hp infection is approximately 30% in developed countries and up to 80% in developing countries [8, 9].

It has been found that the prevalence of Hp infection was relatively high in Cs endemic areas [10]. In vivo experiment domonstrated that the liver fluke infected hamsters had significantly higher Hp infection rate than non-liver fluke infected hamsters, and co-infection with Cs and Hp can aggravate hepatobiliary abnormality and accelerate the fibrogenesis [11]. There is evidence that Hp can be detected in the gut epithelium of O viverrini (a species of the liver flukes), indicating that the liver fluke represents a reservoir of Hp in the biliary system [12]. In addition, it has been reported that liver fluke infections can lead to glomerulopathy in laboratory animal models [13, 14].

Studies on Hp infection and kidney injury revealed that long-term Hp infection increased the antibodies against Hp, promoted the production of IgA_1_ and its underglycosylation, aggravated renal function and caused more severe antigen deposition in IgA [15, 16]. These findings suggest that Hp might be involved in the pathogenesis of IgA nephropathy through inducing strong mucosal immune response. However, to our knowledge, there is no literature on the association between co-infection with Cs and Hp and renal function. Therefore, we conducted a cross-sectional study to examine the association between co-infection with Cs and Hp infection and estimated glomerular filtration rate (eGFR), and gender-related differences were also investigated.

## Methods

### Study area

The study was conducted at the city of Foshan, which is one of the largest cities in Guangdong Province, China, with a population of nearly 8 million. Clonorchiasis is mainly prevalent in China, and the infection rate in Guangdong province is the highest area of China [5, 17]. However, although the people in Foshan city have a traditional habit of eating raw or undercooked freshwater fish, the detection rate of Cs in this area is relatively low. Guangdong Provincial Hospital of Integrated Traditional Chinese and Western Medicine is the major public hospital responsible for the epidemiological investigation, diagnosis and treatment of infectious diseases in this area. In order to improve the detection rate of Cs, since 2018, the Kato-Katz (KK) method has been widely adopted in general health examinations to provide Clonorchiasis surveillance in this area.

### Study population

We screened the subjects aged 18–65 years who were receiving annual health examinations including Kato-Katz (KK) method for Cs infection and 13C-urea breath test (UBT) for Hp infection from the Health Examination Center of Guangdong Integrated Hospital of Traditional Chinese and Western Medicine from January 2017 to December 2018. All subjects were categorized into four groups: (1) co-infection with Cs and Hp group (Hp(+) + Cs(+) group), (2) Cs infection group (Hp(−) + Cs(+)group), (3) Hp infection group (Hp(+) + Cs(−)group), (4) no-infection of Cs and Hp group (Hp(−) + Cs(−) group). All of the participants with Cs and (or) Hp infection were first diagnosed; No participants enrolled in the study received previous treatments for Hp and Cs infection. Participants with any of the following characteristics were excluded from the study: a) history of kidney disease (or GFR < 60 mL/min/1.73 m^2^); b) alcohol consumption of 2 or more drink units per week; c) history of cancer diseases; d) history of viral hepatitis; e) urinary tract infection; f) autoimmune diseases. This study conformed to the Declaration of Helsinki and was approved by the Ethical Committee of Guangdong Provincial Hospital of Integrated Traditional Chinese and Western Medicine. Written informed consent was acquired from all participants.

### Data collection

Trained medical staff used a questionnaire to collect data on age, sex, alcohol intake and histories of hypertension, diabetes, kidney diseases, autoimmune diseases, cancer diseases and viral hepatitis (Additional file 1). The body mass index (BMI) was calculated as weight in kilograms divided by height in squared meters (kg/m^2^). Venous blood samples were collected after an overnight fast of 8–12 h. All blood samples were tested at the laboratory of Guangdong Provincial Hospital of Integrated Traditional Chinese and Western Medicine. Alanine aminotransferase (ALT), aspartate aminotransferase (AST), alkaline phosphatase (ALP), γ-glutamyltranspeptidase (γ-GT), triglyceride (TG), total cholesterol (TC), fasting plasma glucose (FPG), high-density lipoprotein (HDL), low-density lipoprotein (LDL), creatinine (CRE), blood urea nitrogen (BUN), β_2_-microglobulin (β_2_-MB) and uric acid (UA) levels were measured using an Olympus AU-640 autoanalyzer (Olympus, Japan), and routine blood test were measured using Sysmex 2100 whole blood cell analyzer (Sysmex, Japan).

Stool examination by the Kato-Katz (KK) method was used for the diagnosis of Cs infection, which was performed following the WHO protocol [18]. Briefly, the Kato-Katz thick smears were examined under a microscope by experienced technicians. The number of eggs was counted and recorded. The intensity of Cs infection was expressed by eggs per gram of feces (EPG) and classified into three categories according to WHO [19]: light (1–1999 EPG), moderate (2000–3999 EPG), and heavy (≥4000 EPG). ^13^C-urea breath test (^13^C-UBT) method can be diagnosed as Hp infection when δ^13^CO_2_ > 5‰ [20]. Estimated glomerular filtration rate (eGFR) was calculated using the Chronic Kidney Disease Epidemiology Collaboration (CKD-EPI) equation [21].

eGFR = 141 × min (SCr/κ, 1)^α^ × max(SCr/κ, 1)^-1.209^ × 0.993^Age^ × 1.018(if female), where SCr is serum creatinine (mg/dL), κ is 0.7 for females and 0.9 for males, α is − 0.329 for females and − 0.411 for males, age is in years, min indicates the minimum of SCr/κ or 1, and max is the maximum of SCr/κ or 1.

### Statistical analysis

Normal distribution data were expressed as mean ± standard deviation, and ANOVA was used to compare the groups. Median (interquartile range) was used to describe the data with skewed distribution, and Kruskal-Wallis H test was used to compare the groups. Qualitative data are expressed in frequency (percentage) and compared with chi-square test. Ranked data of the groups were compared using Wilcoxon rank sum test or Kruskal-Wallis H test, as appropriate. In addition, multiple linear regression models were performed to estimate the associations between co-infection with Cs and Hp with eGFR (by the forced entry method). Multiple regression models were adjusted as follows: Model 1 was adjusted for age, gender, history of hypertension and history of diabetes; Model 2 was adjusted for Model 1 + BMI, SBP and DBP; Model 3 was adjusted for Model 2 + ALT, AST, ALP, γ-GT, TC, TG, HDL, LDL, BUN, β_2_-MB and UA. All data analyses were conducted using IBM SPSS (version 22.0). A two-tailed t test was employed, using a significant level of 0.05.

## Results

### Clinical and demographic characteristics

**Fig. 1:**
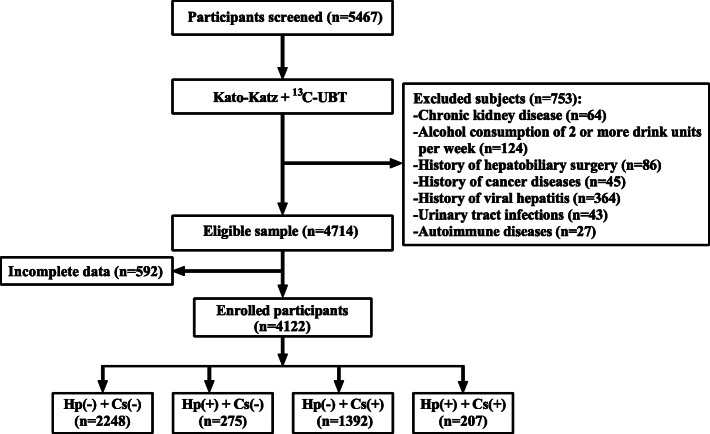
Flow chart of participant selection

Overall, 4122 Chinese participants were enrolled in the study (Fig. 1). The baseline clinical characteristics of the participants were listed in Table 1. Hp infection without Cs infection was present in 6.67% (275/4122) of subjects, while Cs infection without Hp infection was present in 33.77% (1392/4122) of subjects. Co-infection with Hp and Cs were present in 5.02% (207/4122) of subjects. Median age of the participants was 43 years (IQR 35–51). Most of the participants were male (2955/4122, 71.69%). Median eGFR was 96.61 ml/min/1.73 m^2^ (IQR 85.05–106.24). Compared to individuals without Cs and Hp infection, those with co-infection with Cs and Hp were more likely to be older and have higher levels of BMI, γ-GT, TG, FPG, UA, β2-MB, CRE and lower levels of eGFR.

**Table 1:** Baseline clinical characteristics of participants according to the infection of *Helicobacter pylori* and *Clonorchis sinensis*

Parameters	Total (*n* = 4122)	HP(−) + CS(−) (*n* = 2248)	HP(+) + CS(−) (*n* = 275)	HP(−) + CS(+) (*n* = 1392)	HP(+) + CS(+) (*n* = 207)	*p* value*
Age, years	43.0 (35–51)	43.0 (35–50)	44.0 (33–53)	44.0 (36–52)	45 (36–53)	0.01
Gender, male	2955 (71.69%)	1573 (69.97%)	214 (77.82%)	1012 (72.70%)	156 (75.36%)	0.015
BMI, kg/m^2^	23.7 (21.77–25.29)	23.56 (21.60–25.18)	23.78 (21.83–25.29)	23.82 (21.93–26.96)	24.01 (22.04–25.61)	0.007
ALT,U/L	20.0 (15.0–29.0)	20.3 (15.1–29.2)	20.1 (15.0–28.1)	20.0 (15.2–29.0)	14.0 (20.2–24.0)	0.972
AST, U/L	20.0 (17.0–24.0)	20.1 (17.1–24)	20 (18.0–24.2)	20.3 (17.3–24.0)	21.2 (18.0–25.0)	0.858
ALP, U/L	88.35 (81.62–95.58)	88.22 (81.34–94.97)	87.94 (81.31–95.35)	89.35 (82.27–96.49)	87.61 (79.0–95.44)	0.003
γ-GT, U/L	26.0 (19.0–39.0)	25.0 (18.0–38)	27.00 (19–37)	27.00 (19–41)	40.0 (28.0–64.8)	0.002
TG, mmol/L	1.39 (0.95–2.13)	1.36 (0.94–1.36)	1.44 (0.94–2.24)	1.42 (0.97–2.20)	1.49 (1.01–2.14)	0.031
TC, mmol/L	5.27 (4.65–5.97)	5.23 (4.62–5.90)	5.19 (4.59–5.91)	5.36 (4.71–6.06)	5.22 (4.65–6.07)	0.005
FPG, mmol/L	5.05((4.75–5.43)	5.05 (4.74–5.42)	4.98 (4.67–5.81)	5.06 (4.77–5.42)	5.10 (4.85–5.54)	0.028
TBA, μmol/L	3.64 (2.65–4.73)	3.67 (2.73–4.76)	3.46 (2.32–4.58)	3.63 (2.62–4.73)	3.42 (2.0–4.42)	0.041
HDL, mmol/L	1.44 (1.23–1.68)	1.44 (1.23–1.69)	1.40 (1.19–1.69)	1.44 (1.24–166)	1.39 (1.24–1.63)	0.239
UA, μmol/L	390.55 (329.4–460.23)	385.65 (324.75–452.9)	328.6 (391.5–468.5)	335.23 (399.95–466.37)	402.9 (323.1–470.1)	0.006
β_2_-MB, mg/L	1.39 (1.21–1.61)	1.39 (1.21–1.60)	1.40 (1.23–1.63)	1.39 (1.21–1.60)	1.46 (1.29–1.99)	< 0.001
LDL, mmol/L	3.20 (2.68–3.76)	3.18 (2.67–3.73)	3.15 (2.65–3.67)	3.23 (2.71–3.85)	3.17 (2.64–3.78)	0.093
CRE, μmol/L	80.65 (69.1–90.5)	79.65 (67.6–90.1)	83.9 (69.8–91.5)	81.0 (69.5–90.6)	84.4 (77.2–93.1)	< 0.001
SBP, mmHg	119 (111–127.5)	118.65 (111–127)	118.56 (111–127.8)	119.9 (111.9–127.8)	117.9 (109.0–126.8)	0.212
DBP, mmHg	73.11 (68.0–78.49)	73 (68–78)	73 (68.00–78)	74.00 (69.00–79)	72.9 (66–748.3)	0.074
BUN, mmol/L	4.57 (3.88–5.32)	4.54 (3.85–5.3)	4.63 (3.99–5.5)	4.59 (3.9–5.28)	4.67 (3.93–5.49)	0.132
eGFR, ml/min/1.73 m^2^	96.61 (85.05–106.24)	97.18 (85.74–107.02)	94.43 (84.77–104.81)	96.66 (84.53–105.0)	92.22 (79.44–103.75)	< 0.001
Diabetes, n	262 (6.36%)	148 (6.58%)	19 (6.91%)	78 (5.60%)	17 (8.21%)	0.413
Hypertension, n	304 (7.38%)	198 (8.81%)	16 (5.82%)	66 (4.74%)	24 (11.59%)	0.0002

Data are presented as medians (interquartile ranges, IQR) and n (%)

*Cs Clonorchis sinensis*, *Hp Helicobacter pylori*, *BMI* Body mass index, *ALT* Alanine aminotransferase, *AST* Aspartate aminotransferase, *ALP* Alkaline phosphatase, *γ-GT* γ-glutamyltranspeptidase, *TG* Triglyceride, *TG* Triglyceride, *TC* Total cholesterol, *FPG* Fasting plasma glucose, *HDL* High-density lipoprotein, *LDL* Low-density lipoprotein, *CRE* Creatinine, *BUN* Blood Urea Nitrogen, *UA* Uric Acid, *β*_*2−*_*MB* β-2-microglobulin, *eGFR* Estimated glomerular filtration rate

*Comparisons were made among four groups. The χ^2^ test was used for dichotomous variables while the Kruskal–Wallis H test was used for continuous variables

### Association between gender and age-related differences of participants and different intensities of *Clonorchis sinensis* infection

**Fig. 2:**
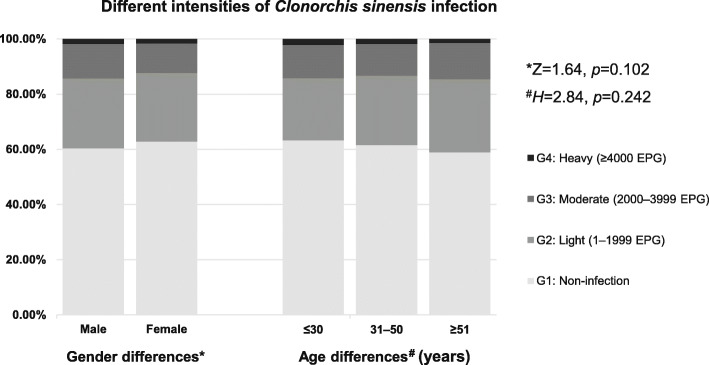
Association between gender and age-related differences of participants and different intensities of *Clonorchis sinensis* infection. For the gender-related groups, in male group, the percentages of G1, G2, G3 and G4 were 60.41, 25.28, 12.49 and 1.83%, respectively; in female group, the percentages of G1, G2, G3 and G4 were 62.81, 24.85, 10.71 and 1.63%, respectively. For the age-related groups, in group A (≤ 30 years), the percentages of G1, G2, G3 and G4 were 63.25, 22.49, 12.07 and 2.19%, respectively; in group B (31–50 years), the percentages of G1, G2, G3 and G4 were 61.55, 25.16, 11.47 and 1.81%, respectively; in group C (≥ 51 years), the percentages of G1, G2, G3 and G4 were 58.94, 26.49, 13.11 and1.47%, respectively

In order to investigate the association between gender and age-related differences and different intensities of Cs infection, all participants were divided into male group and female group according to gender differences, and were divided into three age groups according to age differences (group A: ≤ 30 years; group B: 31–50 years; group C: ≥ 51 years). In addition, participants from gender and age-related groups were subdivided into four grades according to eggs per gram of feces (EPG), namely G1: non-infection, G2: light (1–1999 EPG), G3: moderate (2000–3999 EPG) and G4: heavy (≥ 4000 EPG). The percentages of each grade in the gender and age-related groups were shown in Fig. 2. For the gender-related groups, in the male group, the percentages of G1, G2, G3 and G4 were 60.41, 25.28, 12.49 and 1.83%, respectively; in the female group, the percentages of G1, G2, G3 and G4 were 62.81, 24.85, 10.71 and 1.63%, respectively. There was no significant difference between the gender-related groups with different intensities of Cs infection (*Z* = 1.64, *p* = 0.102). For the age-related groups, in group A (≤ 30 years), the percentages of G1, G2, G3 and G4 were 63.25, 22.49, 12.07 and 2.19%, respectively; in group B (31–50 years), the percentages of G1, G2, G3 and G4 were 61.55, 25.16, 11.47 and 1.81%, respectively; in group C (≥ 51 years), the percentages of G1, G2, G3 and G4 were 58.94, 26.49, 13.11 and 1.47%, respectively. No significant differences were found among the age-related groups with different intensities of Cs infection (*H* = 2.84, *p* = 0.242).

### Comparison of CRE, BUN, β_2−_MB and eGFR of participants with *Clonorchis sinensis* and helicobacter pylori infection

**Fig. 3:**
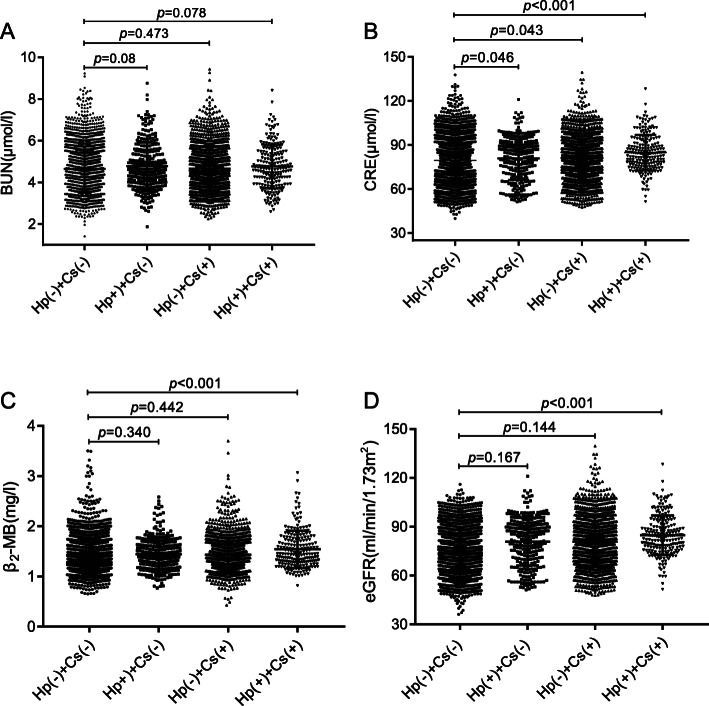
Association between *Clonorchis sinensis* and *Helicobacter pylori* infection and candidate renal function markers (CRE, BUN, β2-MB and eGFR). The levels of CRE, β_2_-MB in co-infection with *Clonorchis sinensis* and *Helicobacter pylori* group were higher and the levels of eGFR was lower than those in non-infection group. In addition, the levels of CRE in infection group were higher than those in non-infection group. However, no differences were found between infection group and non-infection group for BUN

To further investigate the relationship between Cs and Hp infection and candidate renal function markers (CRE, BUN, β2-MB and eGFR). We respectively compared participants infected with Cs or (and) Hp with non-infected participants. As shown in Fig. 3a, for BUN, no differences were found between the infected group and non-infected group. In Fig. 3b, for CRE, there was statistical differences between Hp(+) + Cs(−) group and Hp(−) + Cs(−) group (*p* = 0.046), Hp(−) + Cs(+) group and Hp(−) + Cs(−) group(*p* = 0.043), and the Hp(+) + Cs(+) group and Hp(−) + Cs(−) group (*p <* 0.001). For β_2_-MB(Fig. 3c), significant differences were found between Hp(+) + Cs(+) group and Hp(−) + Cs(−) group (*p <* 0.001), but no differences were found between the Hp(−) + Cs(−) group and the Hp(+) + Cs(−) group (*p =* 0.340) or the Hp(−) + Cs(+) group (*p* = 0.442). For eGFR (Fig. 3d), significant differences were observed between Hp(+) + Cs(+) group and Hp(−) + Cs(−) group (*p* < 0.001). However, no differences were found between the Hp(−) + Cs(−) group and the Hp(+) + Cs(−) group or Hp(−) + Cs(+) group (*p* = 0.167, *p* = 0.144, respectively).

### Comparison of CRE, BUN, β_2−_MB and eGFR of participants with different intensities of *Clonorchis sinensis* infection

**Fig. 4:**
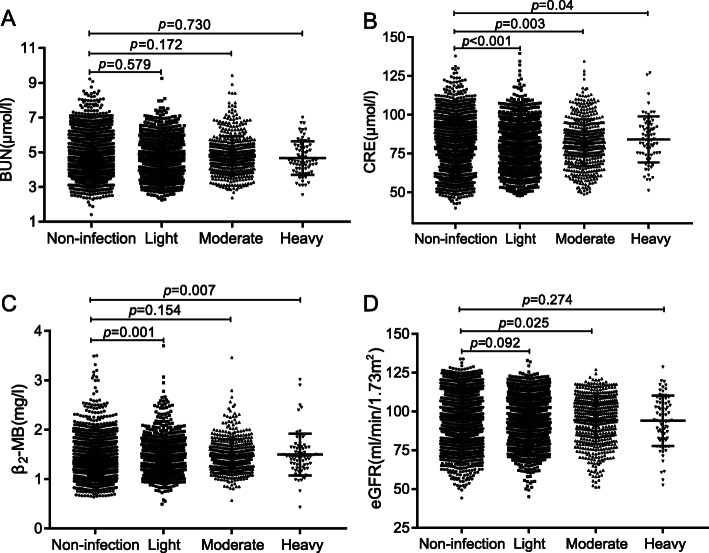
Association between different intensities of *Clonorchis sinensis* infection and renal function markers (CRE, BUN, β2-MB and eGFR). The intensity of *Clonorchis sinensis* infection was expressed by eggs per gram of feces (EPG) and classified into three categories: light (1–1999 EPG), moderate (2000–3999 EPG), and heavy (≥4000 EPG). As shown in Fig. 4, there was no differences between different intensities of infection and non-infection for BUN. The levels of CRE in different intensity infection group were higher and the levels of eGFR in moderate infection group was lower than those in non-infection group. The levels of β_2_-MB in the light and heavy infection groups were higher than those in non-infection group

We also analyzed the association between different intensities of Cs infection and renal function markers (CRE, BUN, β_2_-MB and eGFR). As shown in Fig. 4a, for BUN, there was no differences between infection group and non-infection group (*p* > 0.05). For CRE (Fig. 4b), significant differences were found between the non-infection group and the light, moderate and heavy infection groups (*p* < 0.001, *p* = 0.003, *p* = 0.04, respectively). For β_2_-MB (Fig. 4c), there were significant differences between non-infection group and the light or heavy infection group (*p* = 0.001, *p* = 0.007, respectively), but there were no differences between non-infection group and the moderate infection group (*p* = 0.154). For eGFR (Fig. 4d), significant differences were observed between non-infection group and the moderate infection group (*p* = 0.025). However, no differences were found between non-infection group and the light or heavy infection group (*p* = 0.092, *p* = 0.274, respectively).

### Association between co-infection with *Clonorchis sinensis* and helicobacter pylori and eGFR

In Table 2, the associations among co-infection with Cs and Hp and eGFR were analyzed by multiple linear regression models. In model 1, Hp(+) + Cs(+) was significantly associated with reduced eGFR (β = − 3.08, 95% CI: − 4.63 to − 1.53, *p* < 0.001) after adjustment for age, gender, history of hypertension and history of diabetes. Model 2 (Model 1 + BMI, SBP and DBP) and Model 3 (Model2 + ALT, AST, ALP, γ-GT, TC, TG, HDL, LDL, BUN, β_2_-MB and UA) also showed significant β values. However, there was no association between HP(+) + Cs(−) or HP(−) + Cs(+) and eGFR in the three models. Interestingly, after categorizing subjects by gender differences (Table 3), the relationship remained significant in females but not in males in Model 1 (β = − 10.25, 95% CI: − 12.52 to − 7.98, *p* < 0.001), Model 2 (β = − 9.71, 95% CI: − 11.96 to − 7.47, *p* < 0.001) and Model 3 (β = − 9.37, 95% CI: − 11.60 to − 7.13, *p* < 0.001).

**Table 2:** Association between co-infection with *Helicobacter pylori* and *Clonorchis sinensis* and eGFR

Variables	Model 1^a^			Model 2^b^			Model 3^c^		
	β	95% CI	*p* value	β	95% CI	*p* value	β	95% CI	*p* value
Hp(−) + Cs(−)	reference	─	─	reference		─	reference	─	─
HP(+) + Cs(+)	−3.08	−4.63, − 1.53	< 0.001	−2.81	−4.34, − 1.23	< 0.001	− 1.89	−3.33, − 0.45	0.010
HP(+) + Cs(−)	0.22	−1.16, 1.60	0.757	0.23	−1.14, 1.60	0.738	0.26	−1.02, 1.54	0.692
HP(−) + Cs(+)	−0.14	− 0.88, 0.60	0.713	− 0.03	− 0.76, 0.71	0.945	− 0.01	− 0.68, 0.69	0.990

Abbreviations: *eGFR* Estimated glomerular filtration rate, *CI* Confidence interval, *Hp* Helicobacter pylori, *Cs Clonorchis sinensis*

^a^ Model 1 was adjusted for or age, gender, history of hypertension and history of diabetes. Adjusted R^2^ value is 0.459

^b^ Model 2 was adjusted for Model 1 + BMI, SBP and DBP. Adjusted R^2^ value is 0.469

^c^ Model 3 was adjusted for Model2 + ALT, AST, ALP, γ-GT, TC, TG, HDL, LDL, BUN, β_2_-MB and UA. Adjusted R^2^ value is 0.538

**Table 3:** Association between co-infection with *Clonorchis sinensis* and *Helicobacter pylori* and eGFR in gender difference

Variables	Model 1^a^			Model 2^b^			Model 3^c^		
	β	95% CI	*p* value	β	95% CI	*p* value	β	95% CI	*p* value
**Male**									
Hp(−) + Cs(−)	reference	─	─	reference		─	reference	─	─
HP(+) + Cs(+)	−0.55	− 2.50, 1.40	0.556	− 0.35	(−2.28, 1.58)	0.723	0.68	−1.09, 2.45	0.452
HP(+) + Cs(−)	0.60	−1.10, 2.30	0.492	0.65	(−1.04, 2.34)	0.450	0.72	−0.83, 2.26	0.363
HP(−) + Cs(+)	0.15	−0.79, 1.10	0.750	0.28	(−0.66, 1.21)	0.561	0.48	−0.37, 1.35	0.268
**Female**									
Hp(−) + Cs(−)	reference	─	─	reference		─	reference	─	─
HP(+) + Cs(+)	−10.25	−12.52, −7.98	< 0.001	−9.71	− 11.96, −7.47	< 0.001	−9.37	− 11.60, − 7.13	< 0.001
HP(+) + Cs(−)	−0.75	−2.91, 1.41	0.496	−0.82	− 2.95, 1.32	0.453	−0.69	− 2.79, 1.41	0.521
HP(−) + Cs(+)	−0.81	−1.85, 0.23	0.128	−72	−1.75, 0.31	0.171	−0.83	−1.84, 0.18	0.108

Abbreviations: *eGFR* Estimated glomerular filtration rate, *CI* Confidence interval, *Hp Helicobacter pylori*, *Cs Clonorchis sinensis*

^a^ Model 1 was adjusted for age, gender, history of hypertension and history of diabetes. Adjusted R^2^ values for male and female are 0.317 and 0.540, respectively

^b^ Model 2 was adjusted for Model 1 + BMI, SBP and DBP. Adjusted R^2^ values for male and female are 0.328 and 0.551, respectively

^c^ Model 3 was adjusted for Model 2 + ALT, AST, ALP, γ-GT, TC, TG, HDL, LDL, BUN, β_2_-MB and UA. Adjusted R^2^ values for male and female are 0.438 and 0.568, respectively

## Discussion

To our knowledge, this is the first study to examine the association between Cs and Hp infection with eGFR in the general adult population. The important role of co-infection with CS and Hp in the renal function was highlighted in our study. We found that subjects with CS and Hp co-infection showed relatively reduced eGFR. The association was more significant in females, but not in males.

Most studies on the effects of Cs infection on human were focused on liver and gallbladder diseases [22–24]. Several studies have found that chronic infection with *O.·viverrini* (OV, a species of the liver flukes) may cause hepatobiliary diseases including cholangitis, periductal fibrosis, cholecystitis, obstructive jaundice and cholangiocarcinoma (CCA) [22, 23]. In addition, it has been reported that approximately 10% of clonorchiasis patients are susceptible to CCA [24]. Although renal function is not usually considered in chronic clonorchiasis like many other parasitic infections (e.g. *Plasmodium spp, Schistosoma spp, Filarioidea*) [25], glomerular lesions have been reported in laboratory animal models of OV infection [26, 27]. There is evidence that chronic OV infection may result in significant burden of kidney disease in the form of immune complex-mediated glomerulopathy [28]. Our results showed that there were no significant changes in creatinine and urea nitrogen in Cs*-*infected patients without Hp infection compared with non-infected patients, and there was no correlation with eGFR, which is inconsistent with the above studies [25–27]. We also identified the association between different intensities of CS infection and renal function markers (CRE, BUN, β_2_-MB and eGFR). Our results indicated that there are significant differences between different intensity infection and non-infection participants for CRE (*p* < 0.05) and differences between moderate infection group and non-infection group for eGFR (*p* = 0.025). In addition, significant differences were observed between non-infection group and the light or heavy intensity infection group for β_2_-MB (*p* < 0.01). These findings suggest that patients with different intensities of Cs infection are likely to be associated with impairment of renal function, but its mechanism is not yet clear.

Hp is a spiral-shaped gram-negative bacterium that has been found to naturally colonize the human gastric epithelium. Numerous reports have demonstrated a causal relationship between this infection and chronic gastritis, peptic ulceration, and gastric carcinoma [29, 30]. However, the relationship between Hp infection and kidney injury remains controversial. Recently, a meta-analysis showed that Hp infection might affect the prognosis of kidney diseases, and Hp was the main cause of secondary gastrointestinal diseases for patients with impaired renal function [31]. Several studies suggest that the prevalence of Hp might be lower in long-term dialysis patients than in short-term dialysis patients [32–34]. However, other studies showed that no significant association was found between Hp infection and duration of dialysis [35, 36]. Rasmi et al. [37] found that Hp infection rate was higher in long-term dialysis patients than in short-term dialysis patients. In addition, several studies reported that chronic Hp infection might be a major cause of gastroduodenal and gastrointestinal bleeding in dialysis patients with renal failure [38–40]. Therefore, it can be inferred that Hp infection may directly affect the survival rate of dialysis patients. In addition, there is evidence that long-term infection of Hp infection could increase the antibodies against Hp and aggravate renal function, resulting in more severe antigen deposition in IgA [15]. These findings suggest that Hp might be involved in the pathogenesis of IgA nephropathy through inducing strong mucosal immune response. Similarly, in vitro experiments showed that CagA, a key virulence factor of Hp, may participate in the pathogenesis of IgA nephropathy by influencing the production and glycosylation of IgA_1_ in B cells [16].

Another meta-analysis [41] showed that Type 2 diabetes (T_2_DM) patients with Hp infection had a 2 times higher risk of the occurrence of proteinuria than patients without Hp infection, indicating that Hp infection was associated with the occurrence of proteinuria in T_2_DM patients. Hp radical surgery might be a therapeutic option for protecting renal function in patients with T_2_DM [42]. However, a cross-sectional study [43] investigating the association between Hp infection and chronic kidney disease (CKD) did not find any significant difference in the prevalence of proteinuria and the overall CKD between the Hp infection group and non-infection group. In addition, multiple linear regression analysis showed that the odds of decreased eGFR and proteinuria were still not significantly different between the Hp positive and negative subjects. In the present study, our results showed that whether Hp infection alone, or co-infection with Cs, there was a negative correlation with CRE and eGFR. However, when adjusting for the confounding factors, only co-infection with Cs and Hp had a negative correlation with eGFR. Further subgroup analysis showed that co-infection with Cs and Hp may be associated with reduced renal function in females, but not in males.

Up to now, there was no report about the relationship between co-infection with Cs and Hp and renal function, However, it has been reported that chronic OV infection may result in immune complex-mediated glomerulopathy [28] and Hp might be involved in the pathogenesis of IgA nephropathy through inducing strong mucosal immune response [15, 16]. Therefore, we speculate that co-infection with Cs and Hp is more closely related to renal function impairment. In addition, sex has been viewed as an important factor influencing both renal function and the progression of kidney disease [44]. We thus further hypothesized that there might be sex difference between co-infection with Cs and Hp and renal function impairment. Our results showed that the impairment of renal function was more significant in females, but not in males. It has been proposed that women have more intense immune reactions than men, so they may suffer more immune diseases [45]. In addition, there is evidence that women are more susceptible to immune-induced kidney injury, such as lupus nephritis [46]. However, the specific mechanism still needs further investigations.

We have to note that there are some potential limitations in our research. First, subjects were recruited from the Health Examination Center of our hospital. Most of the participants work in enterprises or institutions with relatively higher education level and economic income, which may not represent the general population. Second, it is a cross-sectional design which makes it difficult to confirm the causality of risk factors. We can only examine the relationship between co-infection with Cs and Hp and eGFR. Third, calculated GFR, but not measured GFR, was used in the study and this indicator was not the gold standard to estimate renal function. Fourth, information regarding medication was not available. Relevant renoprotective medications that block the reninangiotensin-aldosterone system, and potential harmful medications such as nonsteroidal anti-inflammatory drugs, would exert effect on renal function. Fifth, eGFR is a complicated indicator involved in many factors. In addition to hypertension, diabetes and other excluded diseases considered in our study, there are still many factors that may affect renal function. Therefore, our research conclusion is merely a preliminary assessment, and its clinical significance should be analyzed in combination with other indicators in clinical practice. Finally, participants with eGFR> 60 were included in our study, although these differences were statistically significant, the differences in eGFR between the groups were small and may not suggest a clinical difference.

## Conclusion

In this study, our findings suggest that co-infection with Cs and Hp may be associated with reduced renal function in females, but not in males. Nevertheless, the association between co-infection with Cs and Hp and renal function deserves further investigations for the potential pathophysiological mechanisms.

## Supplementary Information

**Additional file 1.**

## Data Availability

The datasets used and/or analysed during the current study are available from the corresponding author on reasonable request.

## Abbreviations

eGFR: Estimated glomerular filtration rate
Cs: *Clonorchis sinensis*
Hp: *Helicobacter pylori*
CKD: Chronic kidney disease
EPG: Eggs per gram of feces
