# Supplementary material for: Association between renal function and co-infection with Clonorchis sinensis and Helicobacter pylori: a cross-sectional study
Source: BMC Infect Dis. 2020 Nov 19;20:868. doi: 10.1186/s12879-020-05616-0 (PMC7678138; doi:10.1186/s12879-020-05616-0)
Supplement: Supplementary file 1 — Additional file 1. [file 12879_2020_5616_MOESM1_ESM.docx]

## **Questionnaire**

The present questionnaire was designed to examine the relationship between co-infection with *Clonorchis sinensis* and *Helicobacter pylori* and renal function. The findings of the study will be general for the study population and will not reflect anything particular of individual person, and your answers will be kept strictly confidential. We will only use the sum of the participants' answers for statistical analysis. You have the right to declare to participate or not in this study. If you decide not to participate, you have the right to withdraw from the study at any time. If you consent voluntarily to be a participant in this study, please sign this form.

Signature of the participant: ________________________

Signature of data collector: __________________________

Interview Code _______________________

Date: ________/_________/_____________ (Day/month/year)

| **Code** | **Variable** | **Response** |
| --- | --- | --- |
| Q001 | Sex | Male €  Female € |
| Q002 | Date of birth | __________ (Day/month/year) |
| Q003 | Marital status | Single or never married €  Married €  Divorced €  Widowed € |
| Q004 | Occupation | Farmers/herdsmen/fishermen €  Merchants/Employers €  Institutions/Civil servants/Government staff €  Student €  Other |
| Q005 | Drinking history (1 unit = 10 grams of pure alcohol) | Never €  Monthly or less €  2 to 4 units a month €  2 to 3 units a week €  4 or more units a week € |
| Q006 | History of eating raw or undercooked freshwater fish | Never eat raw fish or undercooked freshwater fish €  Eat occasionally €  Eat frequently € |
| Q007 | History of liver fluke infection | Infected but untreated €  Infection but cured €  No infection €  Uncertain € |
| Q008 | History of *Helicobacter pylori* infection | Infected but untreated €  Infection but cured €  No infection €  Uncertain € |
| Q009 | Do you have the history of the following diseases? | Viral hepatitis €  Diabetes €  Hypertension €  Hepatobiliary surgery €  Kidney disease €  Cancer €  Autoimmune disease €  Other |
